# Efficacy of 4 % deltamethrin-impregnated collars against canine visceral leishmaniasis across different areas and the sociocultural burden of collar loss in a middle-income country

**DOI:** 10.1016/j.onehlt.2025.101262

**Published:** 2025-10-31

**Authors:** Patricia Sayuri Silvestre Matsumoto, Karla Letícia Seviero Rampazzi, Valéria Medina Camprigher, Helena Hilomi Taniguchi, Virgínia Bodelão Richini Pereira, Roberto Mitsuyoshi Hiramoto, José Eduardo de Raeffray Barbosa, Roldão Antonio Puci Neto, Rodrigo Albergaria Ressio, Luís Fábio da Silva Batista, Luiz Ricardo Paes de Barros Cortez, Khan Rubayet Rahaman, Mathew Novak, José Eduardo Tolezano

**Affiliations:** a Department of Geography & Environmental Studies, Saint Mary's University, Halifax, Nova Scotia, Canada; b Parasitology and Mycology Center, Adolfo Lutz Institute (IAL), São Paulo, São Paulo, Brazil; c Center for Zoonoses Control of Bauru, Health Secretariat of Bauru, Bauru, São Paulo, Brazil; d Regional Laboratory Center II Bauru, Adolfo Lutz Institute, Bauru, São Paulo, Brazil; e Department of Pathology, Medical School, University of São Paulo, São Paulo, Brazil

**Keywords:** Dogs, Canine visceral leishmaniasis, Deltamethrin collars, One health, Loss, Socio, Cultural, Geographical areas

## Abstract

Despite efforts to control visceral leishmaniasis (VL), the disease remains a major burden in low- and middle-income countries. In South America, insecticide-impregnated dog collars help prevent disease transmission, as dogs are the main reservoirs in urban areas. This study evaluated the efficacy of 4 % deltamethrin-impregnated collars (DMC) against canine VL (CVL) over a 24-month period in an endemic area of Brazil. We compared 941 DMC dogs with 1032 control dogs (C) across four geographic areas with similar baseline disease prevalence. The difference between the DMC and C cohorts was statistically significant (*p* < 0.05), and the study achieved an overall efficacy of 63 %, 51 %, 48 %, and 58 % at the first, second, third, and fourth follow-ups, respectively. Among dogs that remained protected, efficacy was 74 %, 67 %, 100 %, and 100 % across the follow-ups, whereas in dogs that lost their collars between follow-ups, efficacy was 45 %, 10 %, 23 %, and − 11 %. Collar loss between follow-ups was associated with a 2.25-fold increase in the odds of CVL (OR 2.25, *p* < 0.05). No statistically significant geographical variation in collar loss was observed, and most losses were potentially preventable. However, infrequently bathed dogs had significantly higher odds of CVL (OR 9.93, *p* < 0.05). These results help demystify sociocultural stigmas related to collar loss and support the development of targeted public health education initiatives. Ensuring collar retention, incorporating owners' cultural behaviors to promote consistent collar use, and integrating educational actions within the One Health and Health Promotion frameworks are crucial to maximizing the success of large-scale dog interventions in public health.

## Highlights

• A two-year cohort study confirms the high efficacy of 4 % deltamethrin-impregnated collars against canine visceral leishmaniasis.
• Collar loss without replacement significantly increases disease incidence.
• Geographical analysis reveals no significant differences in collar loss or the reasons for losses by area.
• Most collar losses are preventable, offering opportunities for One Health strategies.
• Infrequent dog bathing is associated with increased CVL.

## 1 Introduction

Visceral leishmaniasis (VL) is a severe and widespread disease found in various regions worldwide, resulting in death in 95 % of the untreated cases [1]. In South America, it primarily affects canines in urban areas, but humans are occasionally involved in the VL cycle. Brazil accounts for more than 97 % of the reported human cases in South America [2], where t[2]he disease is caused by *Leishmania infantum* and transmitted by sand flies, *Lutzomyia longipalpis* and *Lutzomyia cruzi* (in Mato Grosso do Sul State), recognized as competent vectors; and *Migonemyia migonei* identified as a putative vector of L. *infantum* in some areas of São Paulo state [3].

The main classical symptoms of human cases are fever, anemia, loss of weight and enlargement of spleen and liver [3,4]. The clinical symptoms in dogs include fever, tremors, sweating, progressive weight loss, cachexia, hepatosplenomegaly, onychogryphosis, lymphadenopathy, epistaxis, ocular lesions-uveitis, polyuria, polydipsia, alopecia, dermatitis, ulcers, nodules, papules, hyperkeratinization of the foot pads, depigmentation, lignification, and pyoderma secondary ([[5], [6], [7]]). Asymptomatic dogs represent a challenge to public health as they are considered highly competent to transmit L. *infantum* [8] and often go undetected due to lack of clinical symptoms.

Beyond biological factors, Low and Middle-Income Countries (LMIC) endemic for VL are particularly affected by social determinants of health, that is, non-medical factors that influence health outcomes [9,10]. Conditions in which individuals are born, grow, live, and age, shaped by economic and political systems, contribute to VL outcomes [10]; however, the extent to which social determinants of health influence canine VL (CVL), especially in the context of public health interventions, remains unclear and underexplored.

Since 2016, the Brazilian Ministry of Health has authorized the treatment of CVL using the drug Miltefosine but this is an individual measure and is not included in the national public health program against VL [11]. In addition, several topical insecticides, including collars, spot-on, and sprays, have been tested to prevent sand fly bites [12] or new cases of CVL [[13], [14], [15], [16]]. The efficacy of insecticide collars has been proven highly effective in anti-feeding and/or fast-killing sand flies [17], thereby preventing vector bites, lowering the risk of CVL in non-infected dogs, and limiting its spread from infected dogs [18]. Nonetheless, one of the main challenges in large-scale insecticide collar interventions is the lack of instruction from health professionals to dog owners on proper use or/and inadequate adherence by owners, sociocultural burdens that LMICs engaged in public health interventions continue to face. While few studies investigated the insecticide collar effect in a large number of dogs in at least three follow-ups [13,19], only one tracked the reason for the loss of collars and behavioral practices of the dog owners [20]), and none investigated differences among geographical areas. The present study aims to: (i) evaluate the efficacy of 4 % deltamethrin-impregnated collars over a two-year period compared with control dogs across different areas; (ii) analyze the reasons for collar loss; and (iii) discuss the social and cultural behaviors of the population related to mass collaring programs against VL.

## 2 Materials and methods

### 2.1 One health and health promotion to design the study

Two concepts were fundamental in designing this study: One Health and Health Promotion. The first emphasizes a need for a widening concept encompassing animal and human, biodiversity, ecological systems, climate dynamics, agricultural frameworks, and human sciences [21]. The second defines health as being created and lived daily by people within the settings in which they learn, work, play, love [22,23] and, more recently, interact online. It is the process of enabling persons to increase control over and improve their health. People must be able to recognize and realize aspirations, satisfy necessities, and transform and cope with the environment [22].

Based on these two concepts, we designed this study within a theoretical framework that reflects the practical aspects of our research. We involved an interdisciplinary team of researchers comprising of social scientists, biologists, veterinarians, health agents, health technicians, educators, and information technology specialists. Two groups played a key role in this study: health agents and dog owners. The first actively understands field challenges related to CVL and contributes to health education and transformative public health efforts. The second group is equally important, as the dog's owner decision to retain or remove their dogs' collars directly influences health-disease dynamics. Comprehending the social aspects of a biological process is mandatory when discussing health, as health is not only the health sector's responsibility, going beyond, from healthy lifestyles to well-being [22].

We developed a One Health and Health Promotion approach that considered not only dogs as the primary focus of public health control but also all involved stakeholders, including owners, the environment, health agents, and educational targets. Before each serosurvey, an interdisciplinary educational program was designed for health agents involving a two-day *learning-teaching* course – recognizing that knowledge is a social, interactive process actively constructed rather than passively received – followed by fieldwork to test the survey data collection process. The course covered information on the VL cycle, prevention strategies, proper collar usage, animal well-being, and technology for real-time data collection. From the second serosurvey onward, results from the previous follow-up were presented to highlight the critical role of health agents in implementing this large-scale intervention and to enhance their ability to effectively communicate findings to the community.

Following our educational course, we designed a survey to be collected in the field that considered not only the biological characteristics of dogs but also sociocultural behaviors related to pet ownership and collar usage. Additionally, we integrated an educational component into the data collection process, using each question as an opportunity for *learning-teaching*. This approach reinforced key concepts about the disease cycle, DMC usage, environmental management, and animal health care. Beyond communicating results to the community during serosurveys, we also disseminated findings from each project phase through social media, creating an environment of transparency and trust and reinforcing that participants were fundamental to the intervention and the research. Additionally, we created a virtual space where participants could report any concerns or questions at any time before the serosurveys.

### 2.2 Study area

**Fig. 1:**
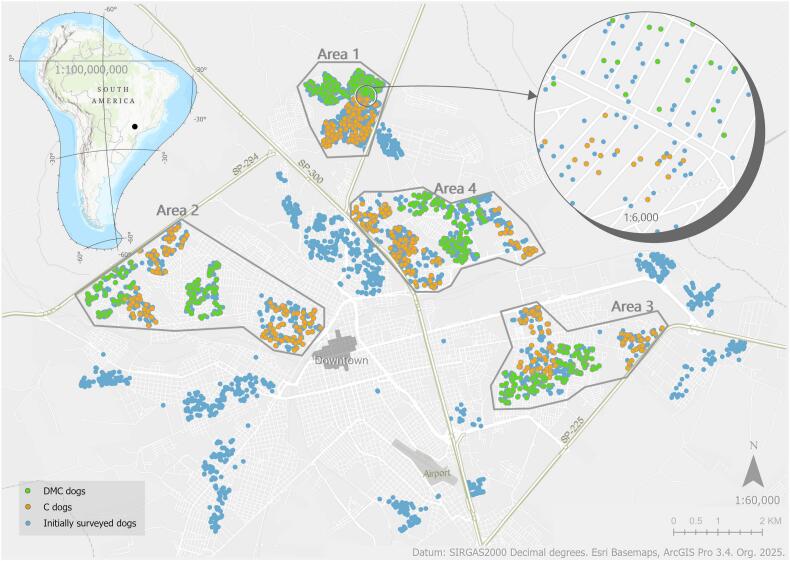
Surveyed dogs (blue), DMC dogs (green) and C dogs (orange). (For interpretation of the references to color in this figure legend, the reader is referred to the web version of this article.) In this cartographic scale, one dot may represent multiple dogs, and the figure does not depict a temporal dimension. All DMC and C dogs were initially surveyed.

Bauru is a municipality located at 22°18′52“ S, 49°03’31” W, in the central west region of São Paulo state, Brazil (Fig. 1). This municipality serves as a key access point to both the capital, São Paulo, and the state of Mato Grosso do Sul (MS). Its population is estimated at 379,297 inhabitants [24] and the dog population at 99,815, following Alves et al. estimation study [25]. Bauru is recognized as an important route for the spread of VL, associated with the expansion of the disease in municipalities from east to west, along major highways [26]. Bauru has been historically recognized for its high incidence of both human and canine cases of VL [27]. As such, the city was chosen to determine the efficacy of 4 % deltamethrin collars in preventing CVL (Scalibor protector band, MSD) [28] in treated deltamethrin collared dogs (DMC) and control dogs (C).

### 2.3 Study cohort design

**Fig. 2:**
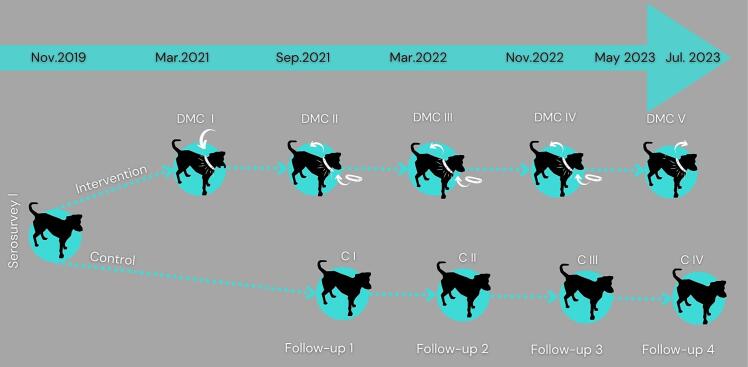
Cohort design: DMC comprises the dogs selected for treatment and C the dog's controls.

The study was conducted from November 2019 to July 2023. An initial serosurvey of stratified household samples was conducted on 6578 dogs to assess CVL prevalence [29]. Next, 2000 dogs were randomly selected within the study area across four geographical areas (Fig. 1). We then divided each area into DMC and C cohorts. Dogs were monitored in four subsequent follow-ups, approximately six months apart (Fig. 2). Deltamethrin collars were placed in DMC I in March 2021 and replaced in the subsequent follow-ups, except in DMC V (Table 1). There was no replacement of collars in case of loss between the serosurveys, and collar loss was systematically recorded by a health agent at each serosurvey.

**Table 1:** Canines recorded in the cohorts and start date.

Cohort	Dogs (n)⁎	Date	Follow-up day
DMC I	941	March 20, 2021	0
DMC II	810	September 18, 2021	180
Control I	1032	November 6, 2021	0
DMC III	732	March 26, 2022	371
Control II	853	May 7, 2022	182
DMC IV	670	November 5, 2022	595
Control III	681	November 26, 2022	385
DMC V	596	May 28, 2023	799
Control IV	478	July 22, 2023	623

⁎ The surveyed canine population includes both the initial participants tracked from the first follow-up and possible newly introduced dogs within the household at the time of blood collection in each cohort.

Serological tests were performed in all the follow-ups, except in DMC I due to the COVID-19 restrictions that were in place at the time. The serosurvey of C cohorts followed that of the DMC cohorts, a strategic decision driven by logistical considerations and the availability of human resources required for the extensive collection of blood and canine data (Fig. 2). New dogs within households were added to the study during the second and subsequent serosurveys; however, no new households were included after DMC I and C I.

### 2.4 Data surveyed

During all follow-ups, general data about dog's identification, well-being, collars usage and a picture of the dog for monitoring purposes were recorded by a health agent at the time of the blood collection (Supplementary Material, Table S1). Data regarding socioeconomic characteristics were recorded in the second follow-up (Table 2).

**Table 2:** Household characteristics in the second follow-up.

Area	Households (n)	% of households	Dogs (n)	% of dogs	dogs per households	Mean monthly income per household (BRL)⁎
Area 1	294	28.02	506	27.13	1.72	3577
Area 2	276	26.31	495	26.67	1.79	3770
Area 3	223	21.25	395	21.28	1.77	3854
Area 4	256	24.40	460	24.78	1.79	3837
Total	1049	100	1856	100	1.76	–

⁎ Brazilian Real currency. At the time of the study, 1 US Dollar (USD) had an exchange rate of 5.06 BRL.

We initially aimed to include 250 dogs for the DMC cohort and 250 for the C cohort, totaling 500 dogs in each area, recognizing the diverse socioeconomic profiles. In the second follow-up, Area 1 recorded a higher number of participant households and dogs due to new dogs rescued or born in the household or puppy dogs not previously surveyed. Mean household income were similar across the areas, with area one recording the lowest mean income. All areas had a similar rate of canines per household, with a mean of 1.76. In the fourth follow-up, additional sociocultural data were collected, such as the dog's bath frequency and wash behavior related to wearing the DMC. Data were collected using tablets (SAMSUNG Galaxy Tab A 32) and uploaded to the Internet in real-time.

### 2.5 Serosurvey

The CVL diagnoses were performed following the protocols of the Brazilian Ministry of Health, which considers a rapid test-dual path platform and, if the result is reagent, it requires an additional Enzyme-linked immunosorbent assay (sensitivity of 94.54 % and specificity of 91.76 %, from Biomanguinhos, RJ, Brazil) [3]. The Center for Zoonoses Control of Bauru and Adolfo Lutz Institute performed the diagnostic assessments during the week following the collection.

To maximize participants' availability, the visits of participant households were conducted on weekends (Saturdays and Sundays) or holidays, and messages advising the day of the visit were sent in advance using the application WhatsApp, which is widely used in Brazil. If the activity was not performed on the first attempt, two more visits were tried after working office hours (between 17:00 and 19:00 h).

### 2.6 Geospacial analysis

Dogs were first geocoded by their household address using a Google API [30]. Next, their coordinates were included in ArcGIS 10.2.2 and later migrated to ArcGIS Pro 3.1.2. Using geoprocessing tools, we randomly selected 250 negative dogs for DMC and 250 for C in each of the four geographical areas (Fig. 1). The database was updated in each project phase with new data and serology diagnoses. New canines at each phase were geocoded using ArcGIS Pro and manually adjusted to ensure the correct location with previous participant dogs of the same household, as geocoding address technique may result in slight positional variations, causing the point location to be a few meters away from the original or subsequent geocoding attempts.

### 2.7 Statistical analysis

Surveys data were collected in Google Forms in real-time, and after tabulating them, statistical analyses were performed in Google Spreadsheets and Microsoft Excel (version 2305). The efficacy of deltamethrin against CVL was calculated according to Eq. (1) [15]:

(1) $$\mathrm{Efficacy}=\left(\frac{I_{c}-I_{\mathrm{DMC}}}{I_{c}}\right)*100$$

Where, I_c_ is the incidence rate of CVL for the control group and I_DMC_ for the DMC. Additional efficacy was calculated by selecting incidence rate of dogs wearing DMC (Yes or No) in the follow-ups; and an analysis of variance (ANOVA) was performed to compare the groups and areas.

Binary logistic regression models were employed to analyze the likelihood of CVL infection based on the use of collars (Yes or No) in the follow-ups and the frequency with which the dogs were bathed (weekly, bi-weekly, monthly, or rarely). In the binary logistic regression models, a value of 1 indicated a positive case and 0 non-case, and 1 represented no collar usage, while 0 collar usage at the moment of the visit (follow-up). The frequencies “weekly,” “bi-weekly,” “monthly,” or “rarely” were assigned 1 or 0 accordingly (e.g., weekly bath, yes = 1, no = 0) and 1 represented a positive case of CVL and 0 a non-case. We accounted for the cases and conditions participating in a follow-up and assessed them in the subsequent follow-up. The statistical analysis was conducted in RStudio v.3, using the `rgdal`, `questionr`, and `oddsratio` packages.

### 2.8 Ethical considerations

Owners who voluntarily chose to participate in this research provided their agreement by signing a Free and Informed Consent, and were informed about potential risks for their dogs, which included allergies or any other complications caused by the usage or ingestion of the insecticide collars [28]. It should be noted that if one dog in the household developed an allergy reaction, all the dogs living there were considered allergic to ensure animal welfare. We selected dogs older than six months to avoid possible false-positive diagnoses in puppies due to antibody responses.

## 3 Results

### 3.1 DMC protection rate and follow-up incidence of CVL

Our study demonstrated an overall efficacy against CVL of 63 %, 51 %, 48 %, and 58 % in the first, second, third, and fourth follow-ups, respectively (Supplementary Materials, Table S2). Notably, among the dogs that consistently wore collars throughout the study period, the efficacy reached 74 %, 67 %, 100 %, and 100 % (Table S3). Conversely, the efficacies for dogs that lost their collars between visits were 45 %, 10 %, 23 %, and − 11 % (Table S4).

**Fig. 3:**
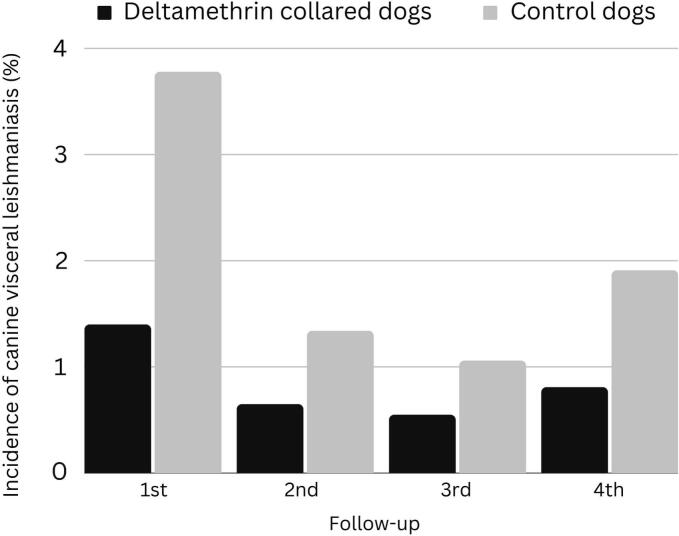
Incidence of canine visceral leishmaniasis in the follow-ups.

Incidence of CVL significantly decreased from the first to the second follow-up in both DMC and C cohorts (Fig. 3), reaching less than 0.9 % in the third follow-up after three DMC replacement cycles except in Area 2 in the C cohort (Table 3). A slightly increase in the incidence rate was observed in the fourth follow-up in the C areas and in DMC area 3.

**Table 3:** Incidence of CVL during the follow-ups per area.

Cohort	Area	Incidence (%)			4rd follow-up
		1st follow-up	2nd follow-up	3rd follow-up	
DMC	Area 1	1.35	0.00	0.81	0.90
	Area 2	1.03	1.05	0.65	0.70
	Area 3	2.60	0.74	0.00	1.90
	Area 4	0.68	0.68	0.67	0.00
	*Total in DMC*	*1.40*	*0.65*	*0.55*	*0.81*
C	Area 1	5.62	0.92	0.00	0.00
	Area 2	4.04	1.57	2.61	3.28
	Area 3	4.76	1.94	0.94	1.61
	Area 4	0.49	1.09	0.72	2.08
	*Total in C*	*3.78*	*1.34*	*1.06*	*1.91*

In the initial follow-up, the incidence of C I (3.78 %) was 170 % higher than DMC II (1.40 %) (*p*-value = 0.0017) (Table S5). Similarly, in the second follow-up, C II (1.34 %) showed a 107 % increase compared to DMC III (0.65 %) (*p*-value = 0.0951). In the third follow-up, the incidence of C III (1.06 %) surpassed DMC IV (0.55 %) by 93 % (p-value = 0.1689). Finally, in the fourth follow-up, the incidence of C IV (1.91 %) was 134 % higher than DMC V (0.81 %) (p-value = 0.0911). No statistically significant differences were observed in the incidence of CVL between follow-ups for DMC (ANOVA p-value = 0.2546) or C (ANOVA p-value = 0.0890) (Table S6, Table S7) nor across different areas for DMC (ANOVA p-value = 0.4343) or C (ANOVA p-value = 0.5031) (Table S8, Table S9). Nevertheless, among the DMC dogs, the subgroup that lost collars more than doubled the incidence of CVL compared to the subgroup that kept their collars; however, statistically significant differences were only observed during the fourth and fifth follow-ups (*p* < 0.05) (Table S10).

### 3.2 Deltamethrin collar losses and sociocultural behaviors

Among 3153 collars placed or replaced in the study (941 in the first follow-up, 810 in the second, 732 in the third, and 670 in the fourth), 20 % (654/3153) were lost. Our binary regression models revealed that dogs losing their collars had 2.25 times higher odds of CVL than those protected (Table 4). No statistical significance was observed across DMC II, DMC III, DMC IV, or DMC V but considering both DMC and C groups, the likelihood of overall DMC was 2.25 and, in the first follow-up, 3.5 (*p*-value<0.05). In other words, losing or non-wearing the collar significantly increased the chance of disease infection.

**Table 4:** Odds Ratios of CVL associated with insecticide collar usage across cohorts.

*Condition*	*OR*	*2.5%*	*97.5 %*	*p-value*
DMC II	1.36	0.43	4.13	0.5833
DMC III	2.76	0.32	23.16	0.3116
DMC IV	1.82	0.33	9.92	0.464
DMC V	NA	NA	NA	0.9945
**Overall DMC (DMC II, III, IV, and V)**⁎	**2.25**	**1.05**	**4.92**	**0.0371**
**DMC II & CI**	**3.50**	**1.70**	**8.47**	**0.0018**
DMC III & CII	2.89	0.78	18.62	0.1657
DMC IV & CIII	1.01	0.26	4.80	0.9885
DMC V & CIV	NA	NA	NA	0.9945
**DMC & C**	**3.48**	**1.97**	**6.72**	**0.0000**

⁎ DMC dogs, placement of DMC I, and collar replacement in the second, third, and fourth follow-ups. Bold is statistically significant (p-value<0.05). NA, Not applicable due to the small sample of infected dogs.

The main reasons for collar loss during the study were dogs removing the collars by themselves (22.48 %), collar removed by the owner due to allergies in the dog (14.71 %), and collar breakage (12.29 %), while 30.57 % of the losses were unaccounted (Table 5). Less frequent reasons included removal for washing or grooming purposes (8.51 %) and collar ingestion (5.46 %). Other reasons, accounting for 5.99 of the losses, include collars were stolen, advised for removal by the veterinarian, collars entangled in objects, or described as ‘had to be’ removed because ‘collar was dirty’, ‘dog gained weight’, and the owner's perception that the dogs exhibited signs of distress.

**Table 5:** Reasons for losing the DMC in the first, second, and third follow-ups.

Area	Allergy	Broken	Eaten	Grooming salon	Unaccounted losses	Removed by themselves	Wash	Total per area	Total per area (%)
Area 1	33	33	9	13	76	55	4	223	24.89
Area 2	31	37	17	10	104	68	5	272	29.10
Area 3	37	27	16	19	69	49	3	220	25.74
Area 4	39	20	10	13	42	42	14	180	20.27
Total per reason	140	117	52	55	291	214	26	952	–
Total per reason (%)	14.71	12.29	5.46	5.78	30.57	22.48	2.73	–	–

Similar rates of collar loss were observed across the four analyzed areas, with no statistically significant differences between them, as shown in Table S11. However, a statistically significant difference was observed in the rates of loss across different follow-up periods (Table S12), with the first follow-up recording a higher rate of losses. Specific reasons for collar loss, such as allergies, baths, grooming salon visits, or self-removal by the dogs, did not show statistically significant variation by area (Tables S13-S16). Nevertheless, Area 2 exhibited a notably higher collar loss rate, especially for unaccounted losses, whereas Area 4 displayed a lower rate. Interestingly, Area 4 also had a higher incidence of collar losses attributed to washing the dog.

Regarding the habit of bathing, out of the 1287 DMC and C dogs included in the fourth follow-up survey, 5.98 % (77/1287) received weekly baths, 33 % (425/1287) bi-weekly, 47.79 % (615/1287) monthly, and 13.21 % (170/1287) rarely. The majority of the dogs (77.65 %; 1001/1287) are bathed only at home, 13.98 % (180/1287) only at the grooming salon, and 8.39 % (108/1287) in both. Among the 788 dogs DMC surveyed in the fourth follow-up, 71.08 % (553/778) were bathed with the insecticide collar, and 28.92 % (225/778) had the DMC removed before washing. DMC dogs bathed weekly had a 0 % incidence of CVL (0/52), dogs bathed bi-weekly 0.40 % (1/249), dogs bathed monthly 0.70 % (3/423) and dogs bathed rarely 1.92 % (2/104). Being bathed weekly, bi-weekly, or monthly was not positively associated with higher odds of CVL cases; however, dogs that were rarely bathed showed a statistically significant increase in disease cases (OR = 9.93, Table 6).

**Table 6:** Odds Ratios (OR) for CVL according to bathing frequency in the fourth follow-up.

*Condition*	*OR*	*2.5%*	*97.5 %*	*p-value*
Bath weekly	0.00	NA	NA	0.9938
Bath bi-weekly	0.71	0.35	5.60	0.7688
Bath monthly	0.30	0.01	2.40	0.3065
**Bath rarely**	**9.93**	**1.17**	**84.36**	**0.0232**
Bath using DMC	1.11	0.14	22.58	0.927
Bath without DMC	0.95	0.04	7.53	0.9682

NA, Not applicable due to the small sample of infected dogs.

## 4 Discussion

Although the evidence supporting the efficacy of 4 % deltamethrin collars against visceral leishmaniasis was established two decades ago [31,32], CVL remains a public health concern in endemic areas. It is well known that insecticide collars have antifeeding [33] and insecticidal effects on sand flies [17], and their use reduces cases of both canine [31] and human VL [34,35]. For this reason, insecticide collars have been pointed out to be a valid measure from the public health perspective [12,16], as they are relatively simple and affordable interventions. In our study, we found an overall protection rate of 63 %, 51 %, 48 % and 58 % in the first, second, third and fourth follow-ups, supporting findings of previous efficacy studies of an overall 58 %, 54 %, and 53 % during follow-up times as calculated by a meta-analysis literature review [16]. Studies with similar sample-size found an effectiveness of 48 % [36] and an estimate of efficacy of 70 % [20]. Efficacy tends to decrease slightly throughout the collaring cycles [16]. From the second cycle onwards, reductions in seroprevalence or incidence can be observed not only in the intervention areas but also in the control areas [37,38], as identified in this study. We observed an abrupt decrease in incidence in the first follow-up and then the incidence rates remained lower than 2 % for both DMC and C, which suggest that DMC has a rapid effect in public health, and an uninterrupted and massive program of collaring dogs would possibly reduce CVL in the entire endemic city.

High efficacy against CVL requires mass use of insecticide collars [39,40] and its continuous usage [36], which is not an easy task as it requires the rapid replacement of lost collars and collaring new dogs that enter the household [31]. We identified that the incidence rate in intervention areas was twice as high among dogs that lost their collars during visits. Moreover, losing or non-wearing the collar was associated with more than twice the odds of disease transmission compared to dogs that remained protected. For protected dogs, the efficacy was 74 %, 67 %, 100 % and 100 % in the follow-ups. Conversely, dogs that lost their collars during the follow-up periods exhibited reduced efficacy of 45 %, 10 %, 23 %, and − 11 %. This suggests that the incidence of unprotected dogs is higher than in control dogs, possibly indicating greater exposure of unprotected dogs in areas of intervention. Such findings suggest that unprotected dogs may attract L. *longipalps*, particularly in environments with extensive protective measures [38].

Most collar losses were either unaccounted for or by deliberate removal by the dog itself, likely due to the fact that most dogs were semi-domiciled, sometimes with unrestricted street access [40], leading to collar loss without retrieval. About 9 % of the collar losses were considered avoidable, for instance, when collars were not replaced after washing or grooming. Surprisingly, the number of collars removed in the grooming salon represented almost 6 % of the losses. The collars have become popular and attractive for the pet industry/commerce, and some clinics/stores can remove them (sometimes on purpose in order to prompt the owner to buy a new one), which will directly impact the dog's protection against CVL. These reasons stress the lack of information about the importance of using the collars as preventative measures despite our efforts to raise awareness of the dog owners since the first follow-up [20].

Moreover, these reasons also give clues for understanding cultural behavior in routine animal health practices. In such endemic places of warmer climates, where the temperature can feel 45 °C, frequent dog washing (at home or in the grooming salon), may be an extension of human bathing culture with Brazil ranking first in the worldwide weekly bathing habits [41]. The manufacturer of the collars recommends an increase of the efficacy 2–3 weeks after placing the collars [28]; however, the effects of shampooing on the duration of efficacy have not been investigated [42]. Surprisingly, we found no increase in the incidence of CVL or higher odds of disease cases for dogs that bathe weekly, bi-weekly, or monthly; on the other hand, we found a statistical association of higher chances of CVL for those who rarely bathe. Given the insecticide components within the sebaceous glands of the skin [43], our results suggest that repellent efficacy may not be impacted by bathing. However, other factors, such as the dog exposure in the peri-domicile, may influence infection status. Dogs that are bathed frequently tend to live indoors, while those that are rarely bathed may spend more time in backyards, potentially at higher exposure to sand flies. These results open a discussion for the sociocultural habits of dog owners in using DMC in their dogs, but further studies are required to investigate these dynamics.

Furthermore, about 6 % of the collars were removed for other reasons, such as ‘they looked ‘dirty’. The collars used in this project were initially white/beige, and after wear and tear, can have an appearance of ‘dirty’, as recorded by the dog's owners. This could be avoided if the collars were fabricated in another color that visually does not ‘look dirty’, for instance, brown or black. Another collar protector brand, imidacloprid/flumethrin, has proven high efficacy (>90 %) [14,15] and the gray collar design, among other factors, may contribute to this result. Cleaning the collars can also impact their efficacy, possibly reducing the repellent effect and/or life span.

The only adverse effect of the collars reported by the dog's owner was dog allergies, which represented an average of 4 % of the collared dogs, similar to other studies of about 5 % [20,36]. Reasons such as being ‘eaten’, ‘broken’, ‘unaccounted losses’, or ‘removed by themselves’ show the collars as an ease-breaking device, mainly for those very playful and lively animals. Dogs that lose their collars shortly after placement or replacement may have been due to having no previous experience of being collared [31], a common socioeconomic and cultural characteristic of dogs in LMIC.

Although the four areas present variations in mean household income [38], no statistically significant differences were observed in collar loss or the reasons for loss among them. This finding deconstructs socioeconomic stigmas, thereby facilitating an overall public health target education program. It is worth noting, however, a significant difference in the number of ‘unaccounted losses’ observed in area 2 (the second lower-income area) and a higher number of collar losses due to washing in area 4 (the second higher-income area). Unaccounted losses may include animals that were frequently observed as free-roaming in Area 2. Loss due to wash reinforce the recommendation of promptly placing collars after washing or avoiding their removal during the bath. However, both scenarios require further investigation.

Finally, limitations of this study should be acknowledged. Blood collection was not performed when the DMC was placed due to COVID-19 pandemic restrictions and, because of this, dogs remained collared for two consecutive cycles. A six-months delay may have limited the number of participants for the final serosurvey, as the canine population is dynamic and in each follow-up, the number of participant dogs tends to decrease. Additionally, although a large number of dogs participated in the efficacy study, some sociocultural variables, such as bathing habits, were only introduced in the fourth follow-up, guided by previous assessments. Nevertheless, these findings provide valuable summary statistics and insights for future research on sociocultural factors affecting DMC adherence. Ultimately, as VL is a heterogeneous disease, other environmental and socioeconomic factors may influence its transmission. Future research should explore how CVL transmission dynamics in relation to DMC use are affected by these broader environmental and social determinants.

## 5 Conclusions

The DMC intervention was highly effective in preventing CVL among dogs that remained collared, while efficacy decreased in those that lost their collars during a large intervention study in Brazil. Insecticide collars had a strong initial effect in reducing CVL incidence at the beginning of DMC cohort and also in control areas during subsequent follow-ups. This finding suggests that massive collaring programs tend to decrease disease incidence over time, maintaining stable low incidence rates and coming close to achieving control when collar retention is high. Collar losses reduce efficacy, increase costs, and pose challenges to implementing insecticide collars as a public health action. Education programs should be implemented for large-scale collaring programs to effectively prevent and reduce CVL in endemic areas of LMIC. Doing so not only promotes the use of insecticide collars but also ensures their proper and consistent use. We recommend integrating educational initiatives alongside public health programs for VL control, incorporating them into primary education, social outreach efforts, and online media campaigns. Future research and public health programs should also prioritize transparency in communicating research findings and disease risks at all levels of analysis to the community, considering health agents and dog owners active participants rather than passive recipients, enabling them to increase control over CVL and improve public health outcomes.

Moreover, we recommend rapid replacement of lost collars. However, due to financial constraints and feasibility concerns, we suggest that collaring efforts focus on adult dogs, as fast-growing puppies may require frequent replacements. Providing dog owners with cable ties for adjusting collars in case of breakage or size changes may also improve retention. Finally, collaboration with veterinary practices could provide an opportunity to educate and inform best practices for collar use within public health programs. However, caution is needed to prevent the unintended commercialization of insecticide collars. Further research should explore the development of more durable collars and examine cultural practices related to canine care and protection in LMIC, including playful behavior, cohabitation with multiple dogs, bathing habits, free-roaming, etc. These factors should be examined not only from an individual animal health care perspective but also within the broader framework of public health. Recognizing insecticide collars as component of the social determinants of health is essential for developing more effective public policies and ensuring sustainable disease control strategies.

## Funding

This research was funded by the 10.13039/501100001807São Paulo Research Foundation (FAPESP): [grant numbers: “2019/22246–8” (PSSM), “2021/08630–0” (PSSM), “2021/03872–5” (KLSR), “2018/25889–4” (JET), “2017/50333–7” (JET), 2024/15388-9 (JET), and the Secretary of Health of São Paulo State FESIMA/GAPS [grant number “2019/01057” (JET)].

## CRediT authorship contribution statement

**Patricia Sayuri Silvestre Matsumoto:** Data curation, Investigation, Project administration, Visualization, Writing – review & editing, Conceptualization, Formal analysis, Methodology, Software, Writing – original draft. **Karla Letícia Seviero Rampazzi:** Formal analysis, Methodology, Validation, Writing – review & editing, Investigation, Software, Visualization. **Valéria Medina Camprigher:** Project administration, Methodology, Writing – review & editing. **Helena Hilomi Taniguchi:** Methodology, Writing – review & editing, Conceptualization, Project administration. **Virgínia Bodelão Richini Pereira:** Conceptualization, Project administration, Writing – review & editing, Methodology, Resources. **Roberto Mitsuyoshi Hiramoto:** Methodology, Writing – review & editing, Conceptualization, Project administration. **José Eduardo de Raeffray Barbosa:** Writing – review & editing, Methodology. **Roldão Antonio Puci Neto:** Methodology, Writing – review & editing, Project administration. **Rodrigo Albergaria Ressio:** Writing – review & editing, Methodology. **Luís Fábio da Silva Batista:** Writing – review & editing, Methodology. **Luiz Ricardo Paes de Barros Cortez:** Methodology, Writing – review & editing. **Khan Rubayet Rahaman:** Writing – review & editing, Methodology. **Mathew Novak:** Methodology, Writing – review & editing, Supervision. **José Eduardo Tolezano:** Formal analysis, Investigation, Project administration, Supervision, Conceptualization, Funding acquisition, Methodology, Resources, Writing – review & editing.

## Declaration of competing interest

The authors declare that they have no known competing financial interests or personal relationships that could have appeared to influence the work reported in this paper.

## Data Availability

Data will be made available on request.

## Appendix A Supplementary data

Supplementary material Image 1
