# Supplementary material for: Efficacy of 4 % deltamethrin-impregnated collars against canine visceral leishmaniasis across different areas and the sociocultural burden of collar loss in a middle-income country
Source: One Health. 2025 Oct 31;21:101262. doi: 10.1016/j.onehlt.2025.101262 (PMC12718145; doi:10.1016/j.onehlt.2025.101262)
Supplement: Supplementary file 1 — Supplementary material [file mmc1.docx]

| **Table S1**: Data surveyed during the blood collection |
| --- |

| **Variable** | **Description** | **Values** |
| --- | --- | --- |
| Address | Location of the household | Text |
| Blood collection date | Date when the blood sample was collected. | Date format (YYYY-MM-DD) |
| Area | Geographic area classification. | 1, 2, 3, or 4 |
| Owner's name | Name of the dog's owner. | Text |
| Owner's cellphone | Contact number of the owner. | String |
| Dog's name | Name of the dog. | Text |
| Dog's ID | Unique identification code for the dog in all serosurveys. | Numeric |
| Dog's evaluation | Health status of the dog at the time of the survey. | Healthy, DMC allergy, Sick, Run away, Died, Donated, Others |
| Wearing the collar | Whether the dog was wearing the deltamethrin collar at the time of evaluation (serosurvey). | Yes / No |
| Reason for not wearing the collar | Justification for collar absence. | The dog has lost it, The collar has broken, The dog had allergies and the collar had to be removed, The dog has eaten it, It was removed in the grooming salon, It was removed by another dog, It was removed by itself, Other (specify) |
| Picture | A picture of the dog at the moment of the serosurvey to ensure data alignment with its characteristics. | JPEG file. |

|  | **Table S2:** Overall efficacy of deltamethrin 4% against canine visceral leishmaniasis | | | | |  |
| --- | --- | --- | --- | --- | --- | --- |
| **1st follow-up** | |  | **Seroconversion I1** | **Seroconversion C** | **Efficacy%** | |
|  |  | Area 1 | 1.33 | 5.62 | 76.33 | |
|  |  | Area 2 | 1.03 | 4.04 | 74.5 | |
|  |  | Area 3 | 2.6 | 4.76 | 45.38 | |
|  |  | Area 4 | 0.68 | 0.49 | -38.78 | |
|  |  | Total | 1.4 | 3.78 | **62.96** | |
|  | |  | **Seroconversion I2** | **Seroconversion C** | **Efficacy%** | |
| **2nd follow-up** | |  |  |  |  |  |
|  |  | Area 1 | 0 | 0.92 | 100 | |
|  |  | Area 2 | 1.05 | 1.57 | 33.12 | |
|  |  | Area 3 | 0.74 | 1.94 | 61.86 | |
|  |  | Area 4 | 0.68 | 1.09 | 37.61 | |
|  |  | Total | 0.65 | 1.34 | **51.49** | |
|  | |  | **Seroconversion I3** | **Seroconversion C** | **Efficacy%** | |
| **3rd follow-up** | |  |  |  |  |  |
|  |  | Area 1 | 0.81 | 0 | NA | |
|  |  | Area 2 | 0.65 | 2.61 | 75.1 | |
|  |  | Area 3 | 0 | 0.94 | 100 | |
|  |  | Area 4 | 0.67 | 0.72 | 6.94 | |
|  |  | Total | 0.55 | 1.06 | **48.11** | |
|  | |  | **Seroconversion I4** | **Seroconversion C** | **Efficacy%** | |
| **4th follow-up** | |  |  |  |  |  |
|  |  | Area 1 | 0.9 | 0 | NA | |
|  |  | Area 2 | 0.7 | 3.28 | 78.66 | |
|  |  | Area 3 | 1.9 | 1.61 | -18.01 | |
|  |  | Area 4 | 0 | 2.08 | 100 | |
|  |  | Total | 0.81 | 1.91 | **57.59** | |

|  |  | | | |
| --- | --- | --- | --- | --- |
| **1st follow-up** |  | **Seroconversion UCI Yes 1** | **Seroconversion C** | **Efficacy%** |
|  | Area 1 | 1.11 | 5.62 | 80.25 |
|  | Area 2 | 0 | 4.04 | 100 |
|  | Area 3 | 3.61 | 4.76 | 24.16 |
|  | Area 4 | 0 | 0.49 | 100 |
|  | Total | 0.99 | 3.78 | **73.81** |
| **2nd follow-up** |  | **Seroconversion UCI Yes 2** | **Seroconversion C** | **Efficacy%** |
|  | Area 1 | 0 | 0.92 | 100 |
|  | Area 2 | 0 | 1.57 | 100 |
|  | Area 3 | 1.02 | 1.94 | 47.42 |
|  | Area 4 | 0.85 | 1.09 | 22.02 |
|  | Total | 0.44 | 1.34 | **67.16** |
| **3rd follow-up** |  | **Seroconversion UCI Yes 3** | **Seroconversion C** | **Efficacy%** |
|  | Area 1 | 0 | 0 | NA |
|  | Area 2 | 0 | 2.61 | 100 |
|  | Area 3 | 0 | 0.94 | 100 |
|  | Area 4 | 0 | 0.72 | 100 |
|  | Total | 0 | 1.06 | **100** |
| **4th follow-up** |  | **Seroconversion UCI Yes 4** | **Seroconversion C** | **Efficacy%** |
|  | Area 1 | 0 | 0 | NA |
|  | Area 2 | 0 | 3.28 | 100 |
|  | Area 3 | 0 | 1.61 | 100 |
|  | Area 4 | 0 | 2.08 | 100 |
|  | Total | 0 | 1.91 | **100** |

**Table S3**: Efficacy of deltamethrin 4% against canine visceral leishmaniasis for dogs wearing collars-Yes

|  | **Table S4:** Efficacy of deltamethrin 4% against canine visceral leishmaniasis for dogs wearing collars-No | | | |
| --- | --- | --- | --- | --- |
| **1st follow-up** |  | **Seroconversion UCI No 1** | **Seroconversion C** | **Efficacy%** |
|  | Area 1 | 1.69 | 5.62 | 69.93 |
|  | Area 2 | 2.86 | 4.04 | 29.21 |
|  | Area 3 | 1.39 | 4.76 | 70.8 |
|  | Area 4 | 2.44 | 0.49 | -397.96 |
|  | Total | 2.07 | 3.78 | **45.24** |
| **2nd follow-up** |  | **Seroconversion UCI No 2** | **Seroconversion C** | **Efficacy%** |
|  | Area 1 | 0 | 0.92 | 100 |
|  | Area 2 | 3.57 | 1.57 | -127.39 |
|  | Area 3 | 0 | 1.94 | 100 |
|  | Area 4 | 0 | 1.09 | 100 |
|  | Total | 1.21 | 1.34 | **9.7** |
|  | | **Seroconversion UCI No 3** | **Seroconversion C** | **Efficacy%** |
| **3rd follow-up** |  |  |  |  |
|  | Area 1 | 1.25 | 0 | NA |
|  | Area 2 | 1.01 | 2.61 | 61.3 |
|  | Area 3 | 0 | 0.94 | 100 |
|  | Area 4 | 0.86 | 0.72 | -19.44 |
|  | Total | 0.82 | 1.06 | **22.64** |
|  | **Seroconversion UCI No4** | **Seroconversion C** | **Efficacy%** |  |
| **4th follow-up** | Area 1 | 2.38 | 0 | NA |
|  | Area 2 | 1.85 | 3.28 | 43.6 |
|  | Area 3 | 4.55 | 1.61 | -182.61 |
|  | Area 4 | 0 | 2.08 | 100 |
|  | Total | 2.12 | 1.91 | **-10.99** |

| **Table S5**: Statistical significance of DMC and C incidence | | |
| --- | --- | --- |
| **Follow-up** | **Condition** | **p-value** |
| 1 | C I > DMC II = 170% | **0.0017** |
| 2 | C II > DMC III = 107% | 0.0951 |
| 3 | C III > DMC IV = 93% | 0.1689 |
| 4 | C IV > DMC V = 134% | 0.0911 |

| **Table S6:** ANOVA for DMC incidence between follow-ups | | |  |  |  |  |
| --- | --- | --- | --- | --- | --- | --- |
| SUMMARY |  |  |  |  |  |  |
| *Groups* | *Count* | *Sum* | *Average* | *Variance* |  |  |
| follow-up 1 | 4 | 5.66 | 1.415 | 0.698967 |  |  |
| follow-up 2 | 4 | 2.47 | 0.6175 | 0.195758 |  |  |
| follow-up 3 | 4 | 2.13 | 0.5325 | 0.131092 |  |  |
| follow-up 4 | 4 | 3.5 | 0.875 | 0.615833 |  |  |
|  |  |  |  |  |  |  |
|  | | | | |  |  |
| *Source of Variation* | *SS* | *df* | *MS* | *F* | *P-value* | *F crit* |
| Between Groups | 1.89725 | 3 | 0.632417 | 1.540929 | 0.254686 | 3.490295 |
| Within Groups | 4.92495 | 12 | 0.410413 |  |  |  |
|  |  |  |  |  |  |  |
| Total | 6.8222 | 15 |  |  |  |  |

| **Table S7:** ANOVA for C incidence between follow-ups | | | | | | | |  | | |  |  |  |
| --- | --- | --- | --- | --- | --- | --- | --- | --- | --- | --- | --- | --- | --- |
| SUMMARY | | |  | | |  | |  | | |  |  |  |
| *Groups* | | | *Count* | | | *Sum* | | *Average* | | | *Variance* |  |  |
| follow-up 1 | | | 4 | | | 14.91 | | 3.7275 | | | 5.075558 |  |  |
| follow-up 2 | | | 4 | | | 5.52 | | 1.38 | | | 0.215133 |  |  |
| follow-up 3 | | | 4 | | | 4.27 | | 1.0675 | | | 1.218625 |  |  |
| follow-up 4 | | | 4 | | | 6.97 | | 1.7425 | | | 1.843892 |  |  |
|  | | |  | | |  | |  | | |  |  |  |
|  | | | | | | | | | | | |  |  |
| *Source of Variation* | | | *SS* | | | *df* | | *MS* | | | *F* | *P-value* | *F crit* |
| Between Groups | | | 17.21127 | | | 3 | | 5.73709 | | | 2.747251 | 0.089053 | 3.490295 |
| Within Groups | | | 25.05963 | | | 12 | | 2.088302 | | |  |  |  |
|  | | |  | | |  | |  | | |  |  |  |
| Total | | | 42.27089 | | | 15 | |  | | |  |  |  |
|  | | |  | | |  | |  | | |  |  |  |
| **Table S8:** ANOVA for DMC incidence between areas | | |  | | |  | |  | | |  |  |  |
|  | |  |  | |  |  |  |  | |  |  |  |  |
| SUMMARY | |  |  | |  |  |  |  | |  |  |  |  |
| *Groups* | | *Count* | *Sum* | | *Average* | *Variance* |  |  | |  |  |  |  |
| Area 1 | | 4 | 3.06 | | 0.765 | 0.3159 |  |  | |  |  |  |  |
| Area 2 | | 4 | 3.43 | | 0.8575 | 0.044892 |  |  | |  |  |  |  |
| Area 3 | | 4 | 5.24 | | 1.31 | 1.351067 |  |  | |  |  |  |  |
| Area 4 | | 4 | 2.03 | | 0.5075 | 0.114492 |  |  | |  |  |  |  |
|  | |  |  | |  |  |  |  | |  |  |  |  |
|  | | | | | | |  |  | |  |  |  |  |
| *Source of Variation* | | *SS* | *df* | | *MS* | *F* | *P-value* | *F crit* | |  |  |  |  |
| Between Groups | | 1.34315 | 3 | | 0.447717 | 0.980571 | 0.434315 | 3.490295 | |  |  |  |  |
| Within Groups | | 5.47905 | 12 | | 0.456588 |  |  |  | |  |  |  |  |
|  | |  |  | |  |  |  |  | |  |  |  |  |
| Total | | 6.8222 | 15 | |  |  |  |  | |  |  |  |  |

| **Table S9:** ANOVA for C incidence between areas | |  |  |  |  |  |
| --- | --- | --- | --- | --- | --- | --- |
| Anova: Single Factor | |  |  |  |  |  |
|  |  |  |  |  |  |  |
| SUMMARY |  |  |  |  |  |  |
| *Groups* | *Count* | *Sum* | *Average* | *Variance* |  |  |
| Area 1 | 4 | 6.54 | 1.635 | 7.245967 |  |  |
| Area 2 | 4 | 11.5 | 2.875 | 1.098167 |  |  |
| Area 3 | 4 | 9.25 | 2.3125 | 2.835425 |  |  |
| Area 4 | 4 | 4.38 | 1.095 | 0.4923 |  |  |
|  |  |  |  |  |  |  |
|  | | | | |  |  |
| *Source of Variation* | *SS* | *df* | *MS* | *F* | *P-value* | *F crit* |
| Between Groups | 7.255319 | 3 | 2.41844 | 0.82881 | 0.503158 | 3.490295 |
| Within Groups | 35.01558 | 12 | 2.917965 |  |  |  |
|  |  |  |  |  |  |  |
| Total | 42.27089 | 15 |  |  |  |  |

|  |  |  |  |  |  |  |  |
| --- | --- | --- | --- | --- | --- | --- | --- |

| **Table S10:** Incidence difference of dogs wearing collars vs dogs not wearing collars. | | |
| --- | --- | --- |
| **Condition** | **Rate** | **p-value** |
| Yes wearing vs No, non-wearing collars — DMC II | No > Yes = 108% | 0.1501 |
| Yes wearing vs No, non-wearing collars — DMC III | No > Yes = 175% | 0.1974 |
| Yes wearing vs No, non-wearing collars — DMC IV | **No > Yes = ∞%** | **0.0403** |
| Yes wearing vs No, non-wearing collars — DMC V | **No > Yes = ∞%** | **0.0216** |

**Table S11**: Overall difference in collar losses by areas

| *Source of Variation* | *SS* | *df* | *MS* | *F* | *P-value* | *F crit* |
| --- | --- | --- | --- | --- | --- | --- |
| Between Groups | 884.1875 | 3 | 294.7292 | 2.7295 | 0.090346 | 3.490295 |
| Within Groups | 1295.75 | 12 | 107.9792 |  |  |  |
|  |  |  |  |  |  |  |
| Total | 2179.938 | 15 |  |  |  |  |

**Table S12:** Overall difference in collar losses by follow-up

| *Source of Variation* | | *SS* | *df* | *MS* | *F* | *P-value* | *F crit* |
| --- | --- | --- | --- | --- | --- | --- | --- |
| Between Groups | 1167.188 | | 3 | 389.0625 | 4.609973 | 0.022848 | 3.490295 |
| Within Groups | 1012.75 | | 12 | 84.39583 |  |  |  |
|  |  | |  |  |  |  |  |
| Total | 2179.938 | | 15 |  |  |  |  |

| **Table S13:** Difference in collar losses due to allergies by areas | | | | |  |  |
| --- | --- | --- | --- | --- | --- | --- |
| *Source of Variation* | *SS* | *df* | *MS* | *F* | *P-value* | *F crit* |
| Between Groups | 13.25 | 3 | 4.4166667 | 0.2319475 | 0.87237375 | 3.490295 |
| Within Groups | 228.5 | 12 | 19.041667 |  |  |  |
|  |  |  |  |  |  |  |
| Total | 241.75 | 15 |  |  |  |  |

| **Table S14:** Difference in collar losses due to removal by themselves by areas | | | | | | |
| --- | --- | --- | --- | --- | --- | --- |
| *Source of Variation* | *SS* | *df* | *MS* | *F* | *P-value* | *F crit* |
| Between Groups | 136.25 | 3 | 45.41667 | 0.81404 | 0.510413 | 3.490295 |
| Within Groups | 669.5 | 12 | 55.79167 |  |  |  |
|  |  |  |  |  |  |  |
| Total | 805.75 | 15 |  |  |  |  |

| **Table S15**: Difference in collar losses due to grooming salon by areas | | | | | | |
| --- | --- | --- | --- | --- | --- | --- |
| *Source of Variation* | *SS* | *df* | *MS* | *F* | *P-value* | *F crit* |
| Between Groups | 4.6875 | 3 | 1.5625 | 0.303644 | 0.822252 | 3.490295 |
| Within Groups | 61.75 | 12 | 5.145833 |  |  |  |
|  |  |  |  |  |  |  |
| Total | 66.4375 | 15 |  |  |  |  |

| **Table S16:** Difference in collar losses due to bath by areas | | | | |  |  |  |
| --- | --- | --- | --- | --- | --- | --- | --- |
| *Source of Variation* | *SS* | *df* | *MS* | *F* | *P-value* | *F crit* |  |
| Between Groups | 13.5 | 3 | 4.5 | 0.8 | 0.5174 | 3.490295 |  |
| Within Groups | 67.5 | 12 | 5.625 |  |  |  |  |
|  |  |  |  |  |  |  |  |
| Total | 81 | 15 |  |  |  |  |  |
